# Treatment of Intrahepatic Cholangiocarcinoma—A Multidisciplinary Approach

**DOI:** 10.3390/cancers14020362

**Published:** 2022-01-12

**Authors:** Felix Krenzien, Nora Nevermann, Alina Krombholz, Christian Benzing, Philipp Haber, Uli Fehrenbach, Georg Lurje, Uwe Pelzer, Johann Pratschke, Moritz Schmelzle, Wenzel Schöning

**Affiliations:** 1 Department of Surgery, Campus Charité Mitte and Campus Virchow-Klinikum, Charité–Universitätsmedizin Berlin, 13353 Berlin, Germany; felix.krenzien@charite.de (F.K.); alina-sophie.krombholz@charite.de (A.K.); christian.benzing@charite.de (C.B.); philipp.haber@charite.de (P.H.); Georg.Lurje@charite.de (G.L.); Johann.Pratschke@charite.de (J.P.); moritz.schmelzle@charite.de (M.S.); Wenzel.Schoening@charite.de (W.S.); 2 Berlin Institute of Health (BIH), 13353 Berlin, Germany; 3 Clinic for Radiology, Charité-Universitätsmedizin Berlin, 13353 Berlin, Germany; uli.fehrenbach@charite.de; 4 Department of Hematology, Oncology and Tumor Immunology, Charité-Universitätsmedizin Berlin, 13353 Berlin, Germany; uwe.pelzer@charite.de

**Keywords:** intrahepatic cholangiocarcinoma, multimodal, liver resection, liver transplantation, neoadjuvant therapy, carcinoma, hepatocellular, liver neoplasms, chemoembolization, therapeutic cholangiocarcinoma, bile ducts, hepatectomy, prognosis, radiofrequency ablation, immunotherapy, functional residual capacity

## Abstract

**Simple Summary:**

This review discusses multimodality treatment strategies for intrahepatic cholangiocarcinoma (iCC). Surgical resection remains the only potentially curative therapeutic option and the central cornerstone of treatment. Adjuvant systemic treatment will be recommended after resection or in the palliative setting. Increasing knowledge of phenotypic subclassification and molecular profiling allows investigation of targeted therapies as (neo-)adjuvant treatment. High-dose brachytherapy, internal radiation therapy, and transarterial chemoembolization are among the interventional treatment options being evaluated for unresectable iCC. Given the multiple options of multidisciplinary management, any treatment strategy should be discussed in a multidisciplinary tumor board and treatment should be directed by a specialized treatment center.

**Abstract:**

Intrahepatic cholangiocarcinoma (iCC) is distinguished as an entity from perihilar and distal cholangiocarcinoma and gallbladder carcinoma. Recently, molecular profiling and histopathological features have allowed further classification. Due to the frequent delay in diagnosis, the prognosis for iCC remains poor despite major technical advances and multimodal therapeutic approaches. Liver resection represents the therapeutic backbone and only curative treatment option, with the functional residual capacity of the liver and oncologic radicality being deciding factors for postoperative and long-term oncological outcome. Furthermore, in selected cases and depending on national guidelines, liver transplantation may be a therapeutic option. Given the often advanced tumor stage at diagnosis or the potential for postoperative recurrence, locoregional therapies have become increasingly important. These strategies range from radiofrequency ablation to transarterial chemoembolization to selective internal radiation therapy and can be used in combination with liver resection. In addition, adjuvant and neoadjuvant chemotherapies as well as targeted therapies and immunotherapies based on molecular profiles can be applied. This review discusses multimodal treatment strategies for iCC and their differential use.

## 1. Introduction

Intrahepatic cholangiocarcinoma (iCC) represents a malignant entity parting from epithelium cells of the intrahepatic bile ducts proximal to the ductus hepaticus dexter, respectively sinister. Defined by the anatomic localization, iCCs are distinguished from perihilar cholangiocarcinomas, distal cholangiocarcinoma and gallbladder carcinoma. Differences in risk factors, histopathologic features, prognosis and applicability of different therapeutic approaches implicate the differentiation of iCCs from other types of cholangiocarcinomas [1]. The clinical relevance of iCC can only in part be based on its epidemiological features. The iCC represents 10–15% of all primary liver cancers [2] and is rare in patients younger than 40 years of age, with the highest incidence between the fifth and seventh decades of life [3]. Geographic differences in incidence are due to underlying risk factors. In Western countries, primary sclerosing cholangitis (PSC) is the most common predisposing factor. In patients with PSC, the annual risk of developing iCC is 0.5–1.5% with a lifetime prevalence of 5–10% [4,5]. Other possible risk factors are infections with the hepatitis B or hepatitis C virus, with particularly high numbers of cases in Asia and Africa, liver cirrhosis, diabetes mellitus or alcohol consumption. In a meta-analysis of 11 studies, the overall odds ratio was estimated to be 5.10 for hepatitis B and 4.84 for hepatitis C [6]. In Southeast Asia, parasitic infection with the hepatobiliary flukes *Opisthorchis viverrini* and *Clonorchis sinensis* is associated with the occurrence of iCC [7,8]. Non-alcoholic fatty liver disease (NAFLD) slightly increases iCC risk [9]. Other risk factors include hepatolithiasis and biliary cysts, including Caroli’s disease and syndrome, although causality between the above risk factors for iCC has not been definitively proven [10,11]. The challenges of clinical management are expressed by a low therapeutic success rate and resulting poor survival outcome. We hereby aim to offer an up-to-date overview of available evidence for interdisciplinary approaches in the treatment of iCC.

## 2. Diagnostics

The iCC is still misdiagnosed in some cases mainly because of similar imaging characteristics to hepatocellular carcinoma (HCC) [12,13]. However, the diagnosis is of paramount clinical importance for the initiation of the appropriate therapy. Screening is not useful for the asymptomatic population due to the low incidence and is not recommended by medical societies [14,15]. In contrast, screening of patients with PSC is recommended due to the significantly increased risk of cholangiocarcinoma. In many centers, patients with confirmed PSC or underlying liver disease are screened among others for iCC using magnetic resonance imaging (MRI) with magnetic resonance cholangiopancreaticography (MRCP) at 6- to 12-month intervals and, in addition, serial determination of the tumor marker Carbohydrate Antigen 19-9 (CA19-9). Screening is often combined with ultrasound, or contrast-enhanced ultrasonography (CEUS) due to its availability, lower cost, and high acceptance [16]. However, the majority of iCC occur without the presence of any risk factors [17]. In this case, either disease-related symptoms or cross-sectional imaging performed for other reasons lead to the diagnosis. The highest sensitivity for staging is provided by MRI, which outperforms computed tomography (CT) in terms of tumor stage and detection of multiple lesions in the liver [18]. Of note, the use of portal venous phase washout instead of conventional washout in gadoxetic acid-enhanced MR imaging prevents misdiagnosis of iCC as HCC in liver cirrhosis [19]. CT and MRI are comparable in detecting lymph node metastases, while sensitivity and specificity remains low [20,21]. In addition, CT is used to assess the anatomy of the liver-supplying vessels before surgery and for thoracic staging and thus complements the MRI diagnostic. In a recent meta-analysis by Lamarca et al. on the use of F-fluorodeoxyglucose positron emission tomography (18FDG-PET) including more than 2000 patients demonstrated a sensitivity of 91.7% but only a specificity of 51.3% for positive lymph node detection [22]. The authors concluded that 18FDG-PET should be included in the current standard of care mainly for the detection of lymph nodes and distant metastases and the detection of recurrence. In addition, 18FDG-PET is controversial in the diagnosis of the primary tumor due to its low specificity and should not be used in a leading role here. In cases of unresectable or borderline iCC, histological confirmation should be performed before initiating therapy, and the tissue obtained should be sufficient for possible molecular pathology (Table 1). Histologic workup is necessary for targeted neoadjuvant, but also adjuvant chemotherapy or immunotherapy. According to the WHO classification (5th edition), iCC can be phenotypically differentiated into small duct type, which shares aetiological, pathogenetic and imaging characteristics with hepatocellular carcinoma and large duct type, which resembles extrahepatic cholangiocarcinoma [23]. The iCC can also originate from the large bile ducts, with columnar, mucinous cholangiocytes, which are also thought to be responsible for the development of precursor lesions (such as intraductal papillary neoplasms). These large duct changes develop mainly in ducts affected by chronic inflammation, as, e.g., in PSC or infection with liver fluke.

## 3. Liver Resection

**Figure 1:**
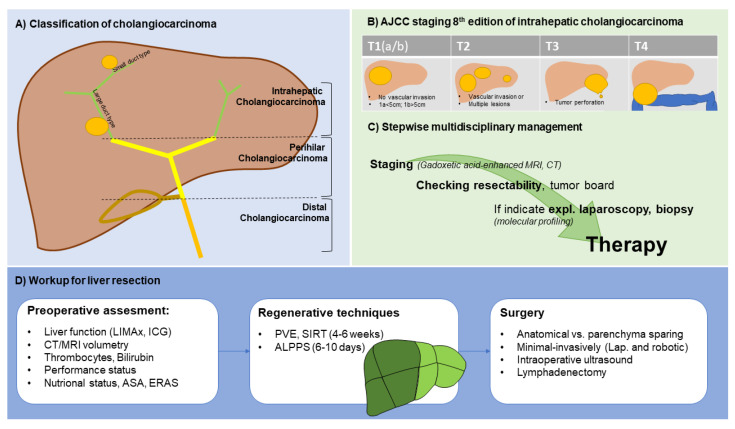
Schematic presentation of staging and management of intrahepatic cholangiocarcinoma.

Liver resection represents the backbone of any curative intended therapy for iCC, while only about 20% of cases are resectable by the time of diagnosis (Figure 1) [24]. The median tumor diameter in surgical cohorts treated was 6 cm, reflecting the often late diagnosis in advanced tumor stages [25]. An analysis of the Surveillance, Epidemiology, and End Results database between 1983 and 2010 confirmed that resections were performed in only 15% of patients with iCC [26]. The therapeutic goal of surgery is complete, margin-negative resection (R0), with the functional residual capacity of the liver and oncologic radicality. These factors represent the determining factors for successful surgery, in addition to the patient’s performance status. Clinical guidelines recommend staging laparoscopy, especially in patients with high CA19-9, risk for occult metastatic disease, vascular invasion, or indirect evidence of peritoneal carcinomatosis with low-grade ascites [27,28]. In an analysis of more than 400 staging laparoscopies in patients with potentially resectable hepatobiliary malignancy, laparotomy was avoided in one of five patients, significantly reducing hospital stay and morbidity [27]. 

Median survival after curative resection is described as 28–30 months and 5-year survival is about 30% [29,30]. Median disease-free survival is calculated to be about 20 months. in contrast, for inoperable cases, the 5-year survival rate is less than 5% [31]. In a multicenter study by Buettner et al. with data from more than 12 HPB centers with more than 1000 patients, the median survival time for a single tumor was 43.2 months, whereas the median survival time for patients with two tumors was 21.2 months; the median survival time for patients with three or more tumors was 15.3 months (*p* < 0.001) [32]. Multiple intrahepatic lesions are associated with a worse prognosis, although resection is the best option for patients even in these cases with survival superior to systemic therapy alone [29,33]. Postoperative morbidity and mortality are important factors in poor postoperative survival, since 75% of iCC cases require extended liver resection [34,35]. A meta-analysis of fifty-seven studies (4756 patients) identified the following high-risk factors for disease recurrence: Lymph node metastases (hazard ratio 2.09), macroinvasion of blood vessels (hazard ratio 1.87), multifocal tumors (hazard ratio 1.70), low histologic grading (hazard ratio 1.5) and tumor size (hazard ratio 1.09) [29]. A multicenter retrospective study from Germany analyzed 156 patients who underwent repeated exploration for recurrent ICC [36]. Median overall survival in the repeat resection group was 65.2 months, with consecutive 1-, 3-, and 5-year survival of 98%, 78%, and 57%, respectively. This enforces the need for discussion of patients with recurrent disease in multidisciplinary tumor boards in presence of an experienced liver surgeon.

In an analysis of 1023 patients with clinically lymph node-positive iCC without extrahepatic involvement from the National Cancer Database, a comparison was made between chemotherapy alone and resection with adjuvant chemotherapy [37]. Patients who underwent resection in combination with chemotherapy had prolonged survival (22.5 months) compared to patients who received chemotherapy alone (11.9 months) or resection alone (12.4 months) (*p* < 0.01). According to an analysis by the Surveillance, Epidemiology, and End Results Program, resection was compared with systemic therapy in 169 patients with lymph node positive iCC without distant metastases [38]. The median survival of patients who underwent surgical resection was not different from that of patients who received chemotherapy alone. However, this study had the limitation that the chemotherapy group was underpowered with only 20 patients. Moreover, after 3 years (36 months), 35% of patients in the resection group were still alive (n = 52), whereas only one patient (n = 1) remained in the chemotherapy group. A beneficial effect of liver resection and chemotherapy compared with chemotherapy alone for positive lymph nodes has been shown in other retrospective cohort studies [39,40].

Resection should merely be performed in the absence of an M1 situation. The oncological advantage of surgical resection over systemic therapy in the presence of distant lymph node metastases has been critically discussed [41,42,43]. Hereby, merely retrospective cohort studies are available and no data are available for resection in combination with adjuvant systemic therapy in this setting. Further investigations are needed including the use of newly available adjuvant treatment options. In case of vascular invasion, highly selected patients might benefit from resection [33,44]. A multi-institutional analysis was performed to investigate the impact of major vascular resections on the outcomes and survival of iCC patients [45]. In 128 of 1087 (12%) patients who underwent resection, major vascular resections were performed. Interestingly, major vascular resection was not associated with an increased risk of complications. Moreover, median recurrence-free (14.0 vs. 14.7 months) and overall survival (33.4 vs. 40.2 months) were comparable in both treatment groups (*p* > 0.05, respectively).

### 3.1. Role of Lymphadenectomy

In general, removal of 6 lymph nodes in the presence of an iCC is required for accurate N staging according to the 8th edition of the American Joint Committee on Cancer staging system [46]. In histologic analyses of iCC patients (n = 4893), positive lymph node invasion was present in 25.2% of cases [47]. Some centers perform routine lymphadenectomies to achieve more accurate staging of nodal status and reduce the risk of local recurrence [48]. Considering the low detection rate on imaging for lymph node involvement [49], the benefits of lymphadenectomy are to be pointed out even more. However, whether lymphadenectomy can improve survival of patients with iCC is not clear and thus still subject of scientific debate [48]. A systematic review by Zhou et al. has investigated whether lymph node resection has an impact on survival. A total of 13 studies were included, comprising 1377 patients [50]. There were no significant differences in overall survival, disease-free survival, or recurrence between lymphadenectomy and no lymphadenectomy, and all studies were retrospective. The authors concluded that lymphadenectomy did not appear to have a positive impact on overall survival and was associated with increased postoperative morbidity. A European multicenter data analysis described an overall survival benefit after resection of ≥3 lymph nodes, although this effect was demonstrated only in patients with lymph node metastases [51]. These results were confirmed in a multicenter retrospective analysis in Korea and Japan [52]. A total of 1138 patients with iCC who underwent liver resection were included. Lymphadenectomy was performed in 413 patients. Surgical removal of more than four (≥4) lymph nodes improved survival outcomes in resected iCC with positive lymph node metastasis (13 months vs. 30 months, *p* = 0.045).

### 3.2. Role of Resection Margins

The European Society for the Study of the Liver guideline defines the goal of surgical therapy for iCC as achieving a microscopic tumor-free resection margin (R0) [14]. R1 resection impairs overall survival and disease-free survival [48]. Interestingly, not only the R1 rate seems to have an impact on survival, but also the margin itself. A meta-analysis from 2016 (cumulative 712 cases) describes a survival advantage for disease-free resection margins > 10 mm [53]. Spolverato et al. published a linear relationship between the size of a clear margin and survival [54]. With increasing safety margin, survival was improved (5-year survival after R1: 13%, 1–4 mm margin: 14%, 5–9 mm: 27%, >1 cm: 32%; *p* = < 0.001; disease-free survival: R1: 9.2 months, R0 > 1 cm 13.2 months). Of note, analyses were performed regardless of lymph node status and N0, N1, and N2 were subsumed. 

Atypical or anatomic resections can be performed for relatively small and peripheral lesions, whereas anatomic resections are usually performed for large possibly even multifocal tumors. Whether anatomic resection is beneficial for survival continues to be investigated, although this consideration is more limited to small peripheral tumors, because the resection line can be chosen more freely. For a retrospective cohort of 702 cases, a slight oncological benefit was shown for anatomical compared to parenchyma sparing resections (5-year-survival: 36% vs. 25.3%; disease free survival: 28% vs. 18%, *p* < 0.05) [55]. However, others were not able to confirm these findings [56]. In summary, a R0 resection should always be aimed at, while in large tumors this is not always possible.

### 3.3. Minimally Invasive Liver Resection

Only a few studies address this issue specifically for the entity of iCC. The small number of publications on minimally invasive liver resection for iCC is not due to its incidence. Indeed, the complexity of hilar lymph node dissection or hepatobiliary reconstruction for extensive tumors has prevented widespread acceptance of minimal invasive techniques for iCC and should be reserved for highly specialized high-volume centers. Nevertheless, in those experienced centers minimal invasive resections of iCC with radical lymphadenectomy are performed with excellent outcomes [57,58]. Further evidence is provided by Ratti et al. who compared 104 open with 104 laparoscopic resections using propensity score matching and found no significant difference in disease-free survival [59]. This has been confirmed in other studies and in a meta-analysis by Machairas et al. [60,61,62]. The authors of this systematic review concluded that laparoscopic liver resection appears to be beneficial for patients with iCC in terms of short-term outcomes, while long-term outcomes of open liver resection and laparoscopic liver resections are comparable. For robotically assisted minimally invasive resections, limited data exist to date specific to the entity of iCC. First data for robotic iCC resections from our center were most recently accepted for publication (Feldbruegge et al., Surg. Endosc). In this paper we report about more than 600 minimally invasive liver resections (25% iCC) and found a 90-day mortality of less than 1% for the robotic group. From our experience, lymphadenectomy in particular is more easily performed when using the robotic approach. Furthermore, positive impact may be inferred based on data on robotic liver resection for other entities [63,64]. Taken together, the minimally invasive approach is supported in guidelines and consensus statements as the preferred technical approach for liver resection whenever technically feasible [65,66].

### 3.4. Management of the Future Liver Remnant

The surgical strategy to increase the rate of liver resections for iCC is to present patients to a designated liver surgery center. If extended liver resection is necessary, preoperative hypertrophy induction should be reviewed. This can be done using future liver remnant volume (FLV) and estimated future liver remnant function (FLRF). The FLV is determined by radiological cross-sectional imaging, the FLRF can be e.g., determined by the Maximal Liver Function Capacity Test (LiMAx Test) or Indocyanine green clearance test (ICG-K) [67,68]. Threshold values for FLR or else FLRF are impacted by possible liver cell damage. An FLR greater than 25% is generally considered sufficient for young patients without liver disease [69]. Inversely, patients with iCC in chronic liver disease require an FLR of more than 40% [70,71].

Several techniques can be used to induce hypertrophy in the FLR. Portal vein embolization (PVE) is performed by percutaneous puncture as well as embolization of the right portal vein system and is now widely accepted as a standard of care [69]. In a healthy liver, sufficient hypertrophy can be observed after two to four weeks, while there are still dropouts due to tumor progression or due to underlying liver disease with lack of hypertrophy [72]. A promising method to improve hypertrophy is to combine PVE with embolization of the right hepatic vein (HVE). This method was evaluated retrospectively and was able to show advantages over PVE alone [73]. Currently, HYPER-LIV01 is ongoing as a randomized controlled prospective trial that is testing PVE against PVE with HVE (NCT03841305) [74]. Note, the HVE may be particularly relevant for liver segment IV, as this is not addressed by classic PVE. Alternatively, the ALPPS procedure (Associating Liver Partition and Portal vein ligation for Stage hepatectomy) may be used. It consists of right-sided portal vein ligation and parenchymal transection in a first step and resection of the tumor after about one to two weeks in a second step after rapid and sufficient hypertrophy. The ALPPS procedure may seem advantageous because the two-stage surgical procedure allows resection after a short period of hypertrophy. There is one RCT (LIGRO trial) for colorectal liver metastases that demonstrated that the intention-to-treat resection rate was 92% (44 patients) for ALPPS compared with 80% (39 patients) for conventional two-stage hepatectomy (*p* = 0.091) [75]. Evidence-based data for ALPPS in iCC are not yet available, while there is evidence for technical feasibility [76]. The ALPPS registry demonstrated that high efficacy can be achieved in R0 resections for locally advanced iCC [77]. Of a total of 102 patients who underwent ALPPS, 99 achieved secondary resection, with a median time between stages of 11 days. The authors compared the surgical approach of ALPPS with chemotherapy alone in the palliative setting. Thus, the comparison with, for example, PVE is not given, and the comparability with other procedures is still pending. Overall, there is insufficient evidence for specific hypertrophy induction, so the specific local conditions in surgical centers are crucial for the choice of hypertrophy induction therapy.

## 4. Liver Transplantation

The experience with liver transplantation in iCC is limited and iCC is a contraindication in most centers worldwide [78]. More commonly, iCCs are often found incidentally in final pathological analysis, for example in patients who have received liver transplantation due to PSC or misdiagnosed HCC in cirrhosis. One of the first reports was published by Pichelmeyer et al., who showed a 1-year survival rate of 53%, although the cohort was very small with 17 patients [79]. A meta-analysis of eight studies with a total of 355 patients yielded pooled 1-, 3-, and 5-year overall survival rates of 75%, 56%, and 42%, respectively [80]. In ‘very early’ iCC (max. diameter up to 2 cm), mostly transplanted with the diagnosis of HCC and in final pathology corrected to iCC, the results are more promising and almost comparable to transplantation for HCC within the Milan criteria [81,82]. These results were similar to a National Cancer Database analysis, which showed that liver transplantation (n = 66) was comparable to liver resection (n = 461) when tumor stage and resection margin were considered [83]. Kaplan–Meier analysis showed a 5-year overall survival rate of 36.1% for patients who underwent liver transplantation compared with 34.7% for liver resection (*p* = 0.53). Liver transplantation is indeed an effective treatment modality in highly selected patients with localized iCC and likely to be evaluated especially in the presence of septal fibrosis or cirrhosis. Recently, Lunsford et al. proposed a protocol for liver transplantation in non-resectable iCC with neoadjuvant chemotherapy [84]. In this regimen, transplantation was performed only if the tumors showed a partial response or at least stable disease after neoadjuvant therapy. Neoadjuvant therapy included gemcitabine-based chemotherapy, such as gemcitabine-cisplatin or gemcitabine-capecitabine, with second- or third-line therapies according to institutional standards. Of 21 included patients, only 6 proceeded to liver transplantation. In this very small group 5-year survival was 83% (five out of six patients) whereas 50% of the transplanted patients developed recurrent disease within 5 years. A multicenter, single-arm, prospective study (NCT02878473) is currently ongoing and recruiting patients for liver transplantation with a single iCC ≤ 2 cm in size, liver cirrhosis, and CA 19-9 ≤ 100 ng/mL. Primary endpoint is 5-year patient survival. In times of ubiquitous organ shortage living liver donation might be a possible option, although ethical considerations have to be made, weighing the donor risk against the overall poor prognosis even after successful transplantation. Another possibility may be the increased use of marginal grafts, especially due to the revival of machine perfusion, that has shown significantly improved outcomes after transplantation of marginal grafts compared to classical cold storage preservation [85]. Despite machine perfusion, more recently, new scores, e.g., the Liver Graft Assessment Following Transplantation (L-GrAFT) algorithm or the Early Allograft Failure Simplified Estimation (EASE) score for the estimation of successful transplants with marginal grafts have been proposed to be helpful in reducing the risk of early allograft failure (EAF) [86,87].

In summary, data for liver transplantation in iCC is limited, despite promising results for a very small subgroup of patients. Patients with cirrhosis and very early stage iCC or highly selected patients with advanced iCC after neoadjuvant therapy may benefit. However, this becomes even more difficult in the light of organ shortage.

## 5. Systemic Treatment

### 5.1. Role for Neoadjuvant Chemotherapy in Intrahepatic Cholangiocarcinomas

As described above, primary surgical resection remains the treatment modality of choice in early stages. Neoadjuvant therapy may be indicated with most evidence in unresectable/borderline resectable iCC to achieve downstage of initially unresectable/advanced tumors [88]. In a multicenter study, 62 cases of iCC received preoperative chemotherapy compared to 995 patients without chemotherapy, describing an equal outcome for overall survival (46.9 months vs. 37.4 months) and disease-free survival (34.1 months vs. 29.1 months), (*p* > 0.05) [89]. It is worth noting that patients receiving preoperative chemotherapy had a more advanced tumor stage with comparable outcome. Consistent with this, a recent study of 169 cases of iCC described a similar outcome in primarily resected cases (n = 137, overall survival of 32.3 months) compared with primarily unresectable cases after neoadjuvant treatment (n = 32, overall survival of 45.9 months) [90]. Interestingly, downstaging and secondary resection was achieved by either chemotherapy alone or selective internal radiation therapy (SIRT) in combination with chemotherapy. Chemotherapy often uses a combination of gemcitabine-cisplatin, based on data from the ABC-02 trial [91], although many other regimens are possible like LV5FU2–cisplatin, capecitabine–cisplatin, cisplatin mono, gemcitabine-oxaliplatin, FOLFIRINOX, capecitabine mono. Few data are available on the success rate of conversion to resectability after neoadjuvant treatment, and results vary widely [92]. A cohort study from 2018 shows a 52% rate of secondary resectability after six months of neoadjuvant chemotherapy (39 of 74 patients) [93]. Further studies show much lower rates of secondary resectability (8 out of 104 and 10 out of 45 cases) [94,95].

Due to the inadequate success of systemic therapy, there is no recommendation for neoadjuvant treatment in resectable iCC. Yadav et al. analyzed a large dataset of the National Cancer Database of the American College of Surgeons and the American Cancer Society of patients with iCC stage I-III [96]. Indeed, neoadjuvant chemotherapy was associated with longer overall survival in patients than in those who underwent upfront surgical resection followed by adjuvant chemotherapy (40.3 months versus 32.8 months; *p* = 0.01). Another cohort study of more than 4000 patients, after propensity matching, demonstrated that neoadjuvant therapy was associated with a 23% reduced risk of death compared with upfront surgery [97]. More data are needed, particularly generated by prospective clinical trials (such as NCT03579771), to define possible indications for neoadjuvant regimens in patients with resectable ICC. 

To date, there are no encouraging data for hepatic artery infusion (HAI) chemotherapy as conversion therapy in unresectable iCC. In a study by Konstantinidis et al., patients with unresectable iCC who received HAI and systemic therapy were compared with those treated with systemic therapy only. While overall survival was significantly improved in patients with the combined approach (30.8 months vs. 18.4 months, *p* < 0.001), conversion or downstaging was achieved in only 8 of 104 patients, allowing secondary resection [94]. 

More recently, specific tumor analysis made it possible to target iCC more precisely. Table 2 offers an overview over ongoing studies that evaluate the use of specific drugs for resectable and unresectable iCCs. PD-1 (Toripalimab), IDH1 (Ivosidenib) and FGFR-2 (Lenvatinib) are among the targeted molecules.

### 5.2. Adjuvant Chemotherapy

**Figure 2:**
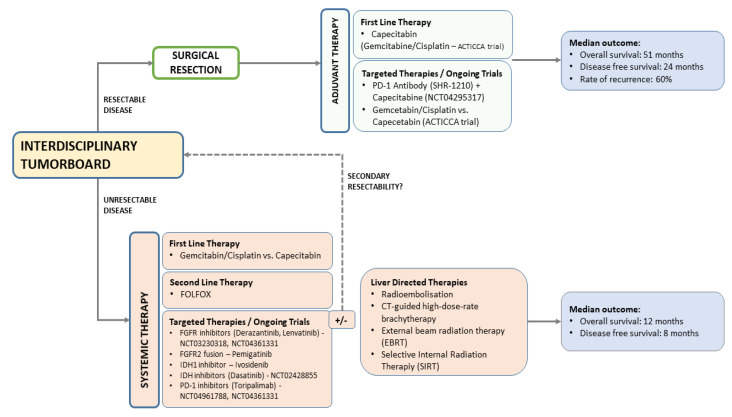
Therapy algorithm of intrahepatic cholangiocarcinoma.

Even after margin-negative resection, the likelihood of recurrence is high. For this reason, there has long been interest in developing effective adjuvant therapies to reduce iCC recurrence (Figure 2). Few studies are available that specifically address the entity of iCC using systemic adjuvant therapies. The PRODIGE trial was a multicenter phase III study and enrolled 196 patients after R0 or R1 resection of localized biliary cancer who received either GEMOX (gemcitabine 1000 mg/m^2^ on day 1 and oxaliplatin 85 mg/m^2^ i.v on day 2 of a 2-week cycle) for 12 cycles or observation [98]. However, there was no significant overall survival benefit after adjuvant treatment with gemcitabine and oxaliplatin (median overall survival 75.8 months) compared with surveillance (median overall survival 50.8 months). The BILCAP trial of cholangiocarcinoma (including gallbladder cancer) treated with capecitabine or observation after R0/R1 resection did not meet its primary endpoint of improving overall survival in the intention-to-treat population [99]. However, the per-protocol analyses suggested that capecitabine may improve overall survival of patients with resected bile duct cancer when used as adjuvant chemotherapy after surgery and may be considered as standard treatment. Indeed, at the end of 60-month follow-up, 56% of patients with cholangiocarcinoma were still alive in the capecitabine group, whereas the survival rate in the observation group was only 41%. A meta-analysis over 15 retrospective studies (n = 5060 patients) analyzed whether adjuvant therapy is beneficial after surgery [100]. Indeed, adjuvant therapy demonstrated superior overall survival (HR = 0.72, *p* < 0.001) but not for disease free survival (*p* = 0.94). These findings led to promoting capecitabine for six months postoperatively as the standard of care in adjuvant treatment of biliary cancer as outlined in the guideline published by the American Society of Clinical Oncology (ASCO) [101]. Furthermore, this caused an amendment in the still recruiting ACTICCA trial [88].

## 6. Interventional Treatments

Only a small proportion of iCCs are resectable at the time of diagnosis, which is why all therapeutic options must be explored in a multidisciplinary tumor board (MDT). Indeed, local ablative treatments are becoming increasingly important in the multidisciplinary management of iCC.

CT-guided high-dose-rate brachytherapy (CT-HDRBT) is one of these therapeutic options. The carcinoma is treated locally with an iridium192 source placed via an afterloading system after puncturing the lesion. Due to its non-thermal mode of action, it can be used for carcinomas of larger diameter and in direct contact with vessels or bile ducts [102]. A retrospective analysis showed a median overall survival of 14 months in 15 patients with primary nonresectable iCC [103]. Long-lasting local tumor control of up to 25 months was achieved. This treatment is linked with a very low incidence of severe complications like hematomas or liver abscesses. Radiation-induced liver disease (RILD), originally described by Ingold et al., can be avoided by careful radiation planning and sparing of non-tumorous liver parenchyma in collaboration with medical physics experts, so that this complication is virtually absent in the setting of CT-HDRBT [104]. However, the indication for this type of therapy is only given in a selected group of patients with sustained liver function (no ascites, no increased total bilirubin [>2.5 mg/dL], no uncorrectable impaired coagulation) and limited disease burden (no multiple/disseminated liver lesions, n > 5).

The option of chemoradiotherapy was investigated in the French phase III FFCD 9902 trial based on a non-inferiority phase II trial [105]. A total of 34 patients were randomized between chemotherapy (gemcitabine/oxaliplatin) and chemoradiotherapy (50Gy with concurrent 5-FU and cisplatin). However, the trial was closed in December 2010 due to slow enrollment before the planned number of patients was reached. This fact again points to the special patient group. According to phase II results, the combination of gemcitabine plus cisplatin was comparable to chemoradiotherapy (50 Gy plus 5 FU and cisplatin) for locally advanced biliary tract cancer. However, study data showed a trend towards chemotherapy alone in progression free survival (11 vs. 5.8 months) and overall survival (20 vs. 13.5 months) [105]. Interestingly, a meta-analysis demonstrated the effect of chemoradiotherapy for the first time [106]. Using a random-effects model, the pooled hazard ratio for overall survival was 0.73 in the chemoradiotherapy group compared with the non-adjuvant therapy group. Nevertheless, it remains unclear whether sequential or concurrent chemoradiotherapy should be performed, which merits further investigation.

Transarterial chemoembolization (TACE) is also discussed as a therapeutic option for inoperable intrahepatic cholangiocarcinomas. To date, TACE has shown mixed study results in patients with unresectable iCC, which may be due to its hypovascular type. In a meta-analysis of five trials, TACE was significantly beneficial in unresectable iCC with a hazard ratio of 0.5 [106]. Li et al. investigated the role of TACE after liver resection for recurrent disease [107]. A total of 122 patients were treated with TACE and 431 without TACE. After propensity score matching, comparable survival rates were observed, although patients in the lowest tertile (nomogram scores ≥ 77) benefited from adjuvant TACE (21.3% versus 6.2% for 5-year overall survival).

Another alternative for non-resectable liver cancers (with liver only disease) may be selective internal radiation therapy (SIRT) with Yttrium-90 radioembolization. In a study of 45 patients with unresectable iCC, SIRT in combination with gemcitabine and/or cisplatin achieved an 18% conversion rate to surgery [95]. Randomized controlled trials are missing so far. A pooled analysis of 12 studies showed a response rate of 28% [108]. Conversion to a resectable situation was achieved in 7 of 73 patients (10%). Overall, survival of patients with iCC after treatment with Yttrium-90 microspheres was comparable to systemic chemotherapy or TACE.

After induction chemotherapy, external Beam Radiation Therapy (EBRT) may be considered in unresectable iCC to improve local tumor control and progression-free survival in the liver and to mitigate tumor-related liver failure [109]. The main limitations of the studies are the lack of patient randomization to evaluate the role of EBRT and missing comparison to other therapies and concomitant selection of patients. In a retrospective analysis of 66 patients with ICC treated with EBRT, a 2-year outcome of 84% local control and 58% overall survival was achieved [110]. Therefore, the American Society for Radiation Oncology typically recommends EBRT after systemic therapy. Adjuvant EBRT is conditionally recommended for resected iCC with high-risk features [109].

The relative rarity and heterogeneity of iCC, and thus the selection of patients for the various interventional therapies, makes it difficult to draw firm conclusions about the efficacy of the different treatments. Further randomized trials with different treatment arms are needed to determine the optimal interventional treatment for inoperable iCC. 

## 7. Targeted Therapies

Over the years, several studies have explored the molecular profiles of iCC, leading to flourishing drug development in this field. As stated above, the recent WHO classification (Fifth edition) includes the differentiation into two histological subtypes “small-duct” and “large-duct” type. As shown in Table 1, these two subtypes can not only be differentiated by etiology and macroscopic features but also by molecular profiles. Especially small-duct iCC shows a high prevalence for markers, e.g., IDH1/2-Mut; FGFR-2- Fusions that can be addressed by targeted therapies. Table 3 shows the prevalence of targeted molecules for small-duct iCCs. 

The most important genetic aberration is the fibroblast growth factor receptor 2 fusion (FGFR2) [111]. Pemigatinib targets this specific fusion; it was approved for the second-line treatment in 2021 by getting the results from the FIGHT202 phase II trial [112]. Median overall survival was 21.1 months with pemigatinib versus 6.9 months with placebo. The FIGHT 302 study is currently recruiting to test the effectiveness of pemigatinib in the first line treatment (NCT03656536). Further projects aiming at FGFR2 are ongoing, for instance the phase III trial for infigratinib (PROOF study, NCT03773302); results are not available yet [113].

Another promising target is isocitrate dehydrogenase (IDH) [111]. In a recently published phase III study (ClarIDHy trial), ivosidenib (AG-120), an inhibitor of mutant IDH1, was compared with placebo in patients with unresectable or metastatic cholangiocarcinoma with IDH1 mutation [114]. Median overall survival was 10.3 months with ivosidenib versus 7.5 months with placebo (hazard ratio 0.79, *p* = 0.09). After adjustment for crossover, the median overall survival with placebo was 5.1 months, reaching significance (*p* < 0.001). The most common grade 3 or higher adverse event was ascites (11 patients [9%] on ivosidenib and 4 patients [7%] on placebo). This study provides efficacy of targeted therapy in iCC and reinforces molecular profiling. Ivosidenib was approved by the FDA in 2021 for second-line treatment in patients with IDH1 Mutation.

In the phase II open-label extension of infigratinib, an objective response rate of 23.1% was observed after a median follow-up of 10.6 months (25 of 108 patients), with only one confirmed complete response in one patient [115]. Other FGFR2 inhibitors under investigation in ongoing Phase III trials include derazantinib and futibatinib [116,117]. In addition, tropomyosin receptor kinases (TRK) inhibitors such as larotrectinib [118] or other targets such as BRAF mutations, NTRK gene fusions or HER2 amplification are being investigated [119].

## 8. Role of Immunotherapies

Various immunotherapeutic drugs continue to be investigated in clinical trials and have not yet been conclusively clarified [127]. In the development of cancer vaccines, the aim is not to prevent the tumor but to actively treat the tumor through an antigen-specific immune response. This has already been investigated in clinical trials, but with little success so far. Dendritic cell (DC)-based immunotherapy targeting synthesized peptides, Wilms tumor 1 (WT1) and Mucin 1, cell surface associated (MUC1) was studied in 65 patients with unresectable, recurrent, or metastatic cholangiocarcinoma [128]. Overall, 0 of 65 patients showed a complete response according to RECIST criteria three months after the first vaccination, with a rate of progressive disease of 6% and stable disease of 23%. In another study, the clinical use of a dendritic cell vaccine plus activated T cell transfer after liver resection of iCC was investigated. A total of 36 patients were inoculated with autologous dendritic cells pulsed from tumor lysate plus ex vivo activated T cell transfer [129]. Indeed, progression-free survival and overall survival were improved with 18.3 and 31.9 months in patients receiving adjuvant immunotherapy versus 7.7 and 17.4 months in the surgery-only group (*p* < 0.05). The use of chimeric antigen receptor (CAR) T cell therapy is also being investigated for iCC, but toxicity requires further investigation and currently only phase I/II results are available [130,131]. The potential of CAR T cell treatment is evident, but more studies are needed to develop further evidence for iCC. 

Blockade of the immune checkpoint with monoclonal antibodies has led to a paradigm shift in a variety of malignancy therapies [132]. A phase 2 study evaluated the clinical activity of pembrolizumab, a humanized immunoglobulin G4 (IgG4) κ anti-PD1 mAb, against programmed cell death protein 1 (PD-1) in 41 patients with advanced metastatic carcinoma with or without mismatch repair deficiency, a number of them having cholangiocarcinoma [133]. The objective response rate and immune-related progression-free survival were 40% and 78%, respectively, demonstrating that mismatch-repair status predicted clinical benefit of immune checkpoint blockade with pembrolizumab. Pembrolizumab has also been studied for advanced bile duct cancer in the KEYNOTE-028 (n = 23, NCT02054806; phase 1b) and KEYNOTE-158 (n = 104, NCT02628067; phase 2) trials [134]. In the KEYNOTE-028 trial, the objective response rate was 13.0% and median overall survival and progression-free survival were 5.7 and 1.8 months, respectively. Pembrolizumab showed durable antitumor activity and few side effects in 6% to 13% of patients regardless of PD-L1 expression. In the KEYNOTE-158 trial, the objective response rate was 5.8%; and overall survival and progression-free survival were 7.4 and 2.0 months, respectively. In PD-L1 expressors (n = 61) and PD-L1 non-expressors (n = 34), the objective response rate was 6.6% (4/61) and 2.9% (1/34), respectively. In a phase II clinical trial of nivolumab in patients with advanced bile duct cancer, the objective response rate was 11%, including a single case of unconfirmed partial response [135]. In the intention-to-treat population, median progression-free survival was 3.68 months and median overall survival was 14.24 months. 

Gemcitabine in combination with cisplatin is the standard systemic treatment (ABC-01 trial), although this therapy is not curative. Therefore, attempts are being made to combine chemotherapy with immunotherapy. Preliminary data from the BilT-01 study have already been published as an abstract. A total of 71 patients were assigned to combinational immunotherapy using nivolumab with gemcitabine/cisplatin (n = 35), or nivolumab with ipilimumab (n = 36) [136]. Median progression-free survival was 8.8 months in Arm A and was higher than in Arm B at 4.1 months. Further follow-up is required. Another phase II study (NCT02829918) also demonstrated that nivolumab was well tolerated and moderately effective with durable responses in patients with refractory biliary tract cancer [135]. Interestingly, there is evidence that the PD-1 inhibitor nivolumab can resensitize biliary tract cancer to chemotherapy with gemcitabine and cisplatin. In a phase II study, patients treated primarily with gemcitabine- or cisplatin-based chemotherapy were subsequently treated with nivolumab plus gemcitabine and cisplatin [137]. Some patients showed a complete response, and one showed a partial response, suggesting that nivolumab is able to resensitize chemotherapy with gemcitabine and cisplatin.

## 9. Conclusions

The available therapies for iCC are subject to constant innovative change. Accurate diagnosis for staging and treatment planning is essential. Liver resection remains the backbone of any potentially curative treatment and is now possible even in advanced tumor stages [138]. It has gained importance in recent years, especially due to decreased morbidity and mortality driven by minimally invasive techniques and the possibility of multimodal approaches (e.g., with the use of preoperative induction of hypertrophy of critical future liver remnants). Decisive for the patient is the discussion of the case at a surgical center with high expertise and in a multidisciplinary tumor board (MDT). In cases of recurrent liver disease, re-resection should be considered. Liver transplantation may be possible under trial conditions or in super-selective cases (small tumors < 2 cm) and the presence of liver cirrhosis, but cannot be generally recommended. Neoadjuvant strategies should only be used when resection is not primarily possible and include chemotherapy as well as interventional procedures. It is important to re-discuss unresectable cases on a regular basis in the MDT, as downstaging is possible in some patients and secondary resectability may be achieved. Data on neoadjuvant therapy for primary resectable iCC are insufficient to date and need further investigation. The results do not yet provide sufficient evidence for efficacy in iCC for immunotherapies, however, chemotherapy and immunotherapies could act synergistically. Importantly, iCC should be molecularly profiled, because in about 25% of cases genetic alterations are present that may be usable for targeted therapies.

## Figures and Tables

**Table 1:** Phenotypic classification of intrahepatic cholangiocarcinoma according to Banales et al. [11].

	Small Duct Type	Large Duct Type
*Morphology*	Mass-forming	Periductal (±mass-forming) or intraductal growing
*Histology*	Small, tubular or acinar adenocarcinoma with nodular growth, invasive into liver parenchyma and minimal mucin production	Large intrahepatic bile ducts, mucin-producing columnar tumor cells arranged in a large ductal or papillary architecture
*Precancerous lesions*	None	Biliary epithelial neoplasia, IPNB, ITPN, mucinous cystic neoplasm
*Predisposing Diseases*	Hepatitis, cirrhosis	PSC, biliary helminthosis, concrements
*Mutations* *Fusions* *Amplifications*	BAP1, BRAF, ARID1A, KRAS, TP53, SMAD4, IDH1/2, FGFR2 fusion	BRCA-1/2-Mut; Her-2-Amp; MSI-high

**Table 2:** Ongoing trials for adjuvant and neoadjuvant therapy.

Trial ID	Protocol	Status	Estimated Enrollment	Estimated Study Completion
NCT04361331Huang XiaoyongShanghai	Toripalimab (PD1)+Lenvatinib vs. Gemox+Lenvatinib in for nonesectable intrahepatic cholangiocarcinoma	recruiting	60	December 2021
NCT02170090 University Medical Center Hamburg, Germany	Adjuvant Chemotherapy With Gemcitabine and Cisplatin Compared to Standard of Care After Curative Intent Resection of Biliary Tract Cancer (ACTICCA-1)	recruiting	781	April 2022
NCT03230318 Mayo Clinic Phoenix, Arizona, United States	Derazantinib in Subjects With FGFR2 Gene Fusion-, Mutation- or Amplification- Positive Inoperable or Advanced Intrahepatic Cholangiocarcinoma	recruiting	143	June 2022
NCT04057365 Massachusetts General Hospital, USA	Study of the Combination of DKN-01 and Nivolumab in Previously Treated Patients With Advanced Biliary Tract Cancer (BTC)	recruiting	30	August 2022
NCT04961788 Shanghai Zhongshan Hospital	Anti-PD1 Antibody Toripalimab Combined With Gemox as First-line Therapy in Late-stage Intrahepatic Cholangiocarcinoma	recruiting	30	December 2022
NCT04961788; Shanghai Zhongshan Hospital	PD1 Antibody (Toripalimab), GEMOX and Lenvatinib vs. no neoadjuvant chemotherapy for resectable intrahepatic cholangiocarcinoma With High-risk Recurrence Factors	recruiting	128	August 2023
NCT05052099 University Hospital, Essen Germany	Phase Ib/II Single-arm Study of mFOLFOX6, Bevacizumab and Atezolizumab in Advanced Biliary Tract Cancer (COMBATBIL)	recruiting	35	June 2024
NCT04989218 University of Alabama at Birmingham	Durvalumab and Tremelimumab With Platinum-based Chemotherapy in Intrahepatic Cholangiocarcinoma (ICC)	not yet recruiting	20	October 2024
NCT04301778 Sidney Kimmel Comprehensive Cancer Center Baltimore, United States	Durvalumab in Combination With a CSF-1R Inhibitor (SNDX-6532) Following Chemo or Radio-Embolization for Patients With Intrahepatic Cholangiocarcinoma	recruiting	30	September 2025
NCT03673072 Krankenhaus Nordwest, Frankfurt Germany	Neoadjuvant Chemotherapy With Gemcitabine Plus Cisplatin Followed by Radical Liver Resection Versus Immediate Radical Liver Resection Alone With or Without Adjuvant Chemotherapy in in Front of Radical Resection of BTC (GAIN)	recruiting	300	November 2024

**Table 3:** Molecular alterations in small duct iCC.

	Molecular Alteration	Frequency (%)	Reference
Authorized targeting drug available	FGFR2 translocation	15–18	Komuta et al. [120]
	IDH1/2	10–15	Javle et al. [115]
	BRAF V600E	3–6	Hyman et al. [121]
	ERBB2	2–3	Oh and Bang et al. [122]
	MSI-H	1–2	Le et al. [123]
	NTRK1-3	<1	Kam et al. [119]
	NRG1	<1	Kam et al. [119]
Authorized targeting drug not available	TP53 mutation	20–30	Thornblade et al. [124]
	RAS mutation	10–20	Thornblade et al. [124]
	ARID1A	5–15	Bezrookove et al. [125]
	BAP1	5–15	Moshbeh et al. [126]
